# Probiotics on chronic urticaria: A randomized clinical trial study

**DOI:** 10.22088/cjim.14.2.192

**Published:** 2023

**Authors:** Abbas Dabaghzadeh, Javad Ghaffari, Siavash Moradi, Davood Sayadian Separghan

**Affiliations:** 1 Pediatric Infectious Diseases Research Center, Mazandaran University of Medical Sciences, Sari, Iran; 2 Molecular and Cell Biology Research Center, Faculty of Medicine, Mazandaran University of Medical Sciences, Sari, Iran; 3 Community Medicine Specialist, Education Development Center, Mazandaran University of Medical Sciences, Sari, Iran

**Keywords:** Chronic urticaria, Histamine antagonists, Probiotics, Therapeutics

## Abstract

**Background:**

Urticaria is a common itchy skin condition characterized by swelling and erythema. A variety of treatments is available today. The purpose of this study was to evaluate the clinical effects of probiotic use in patients with chronic resistant urticaria.

**Methods:**

This four-way blind randomized clinical trial was conducted between June 2019 and June 2020. Study population consisted of patients with chronic urticaria who did not respond well to first line treatment with antihistamines. For the intervention group, antihistamine (cetirizine) and probiotics (femilact capsule) and for the control group, antihistamine (cetirizine) and placebo were administered twice a day for 8 weeks. The “Urticarial Activity for 7 Days” (UAS7) questionnaire was used to assess urticaria activity and the Dermatology Life Quality Index (DLQI) questionnaire was used to assess the quality of life of patients.

**Results:**

Patients’ age range was 7 to 30 years with a mean and standard deviation of 23.6±9.2 years. 31 (81.57%) cases were females and 7 (18.42%) cases were males. Twenty patients were in the intervention group and eighteen patients were in the control group. The mean scores of UAS7 questionnaire were reduced in both groups, but it was more significant in the intervention group (9.6±6.4) compared to the control group (12.7±8.1) at the end of week eight of treatment (P=0.036). Also, there was no significant difference in the quality of life between the two groups after 8 weeks (P=0.805).

**Conclusion:**

This study showed that probiotic consumption along with antihistamines significantly improved the activity of urticaria but not the quality of life of patients.

Urticaria or hives is a pruritic cutaneous disorder with central edema (wheal) and peripheral erythema (flare) (1). The prevalence of urticaria is 10-30% in the general population (1, 2). The exact etiology of urticaria is unknown, but genetic and environmental factors contribute to it (3). Chronic urticaria (CU) which lasts for more than 6 weeks, is less common than acute urticaria. Urticaria can be spontaneous or inducible, although its etiology is unknown in 80-90% of CU cases. Of these patients with unknown etiologies, 40-50% have autoimmune pathophysiology (such as IgG against IgE or IgE receptor; FCƐR1) and 40-50% are purely idiopathic (1, 2, 4). Aeroallergens (indoor and outdoor) can trigger chronic urticaria. In the North of Iran, mite was the most common positive in CU patients (5-7). Helicobacter pylori might be a risk factor for inducing urticaria (8). Malignancies rarely induce acute or chronic urticaria (9). Urticaria, both acute and chronic, especially chronic, has significant effects on patients’ quality of life (10, 11). Acute urticaria often presents due to infections, drugs, foods and insect bites (1, 2). Diagnosis of urticaria is often clinical (careful history and physical examination). In special conditions it might be necessary to run laboratory tests. In acute urticaria, there is usually no need for laboratory examination (1, 2, 12). 

Avoidance of known triggering agents or treatment of a known underlying disease is the most important treatment. The first line pharmacological treatment of urticaria is antihistamines (AHs). Most cases of urticaria improve with AHs which are safe and have few complications. Second generation antihistamines are preferred because they are less sedating and more effective. Second line of treatment is doubling the dose of AH. Third line treatment includes anti-leukotrienes and omalizumab (150 or 300 mg every 4 weeks) (1, 2, 13-17). Probiotics are live microorganisms that are useful for repairing the host’s beneficial microbial flora in the gastrointestinal tract when administered in adequate amounts. Probiotic microorganisms that are widely used include Lactobacillus acidophilus, Lactobacillus Bulgaricus and Bifidobacterium Bifidum (18, 19). 

Microbial agents contribute to allergic disorders such as urticaria. One study reported change in gastrointestinal microbiota of CU patients compared to the general population and Lactobacillus and Bifidobacterium numbers were significantly reduced. Microbiota imbalance can result in susceptibility to urticaria and it is suggested that allergic or inflammatory diseases can be prevented or improved with the addition of lactic acid to patients’ diet. (20-21). In our research, there were few articles studying the effects of probiotics on CU; for example, Nettis et al. report that the combination of Lactobacillus salivarius LS01 and Bifidobacterium breve BR03 improves symptoms of chronic spontaneous urticaria (CSU) (22). 

Different strains and colonies can have different effects on the intestinal flora and resolution of CU. We found a few studies on probiotic effects on CU. There is a need for non-synthetic substances that are also safe for the treatment of CU. There is a gap in research on the effects of probiotics in treatment of CU. The aim of this clinical trial was to document the effects of probiotics (extended strains compare to other studies) on CU patients. 

## Methods

**Figure 1:**
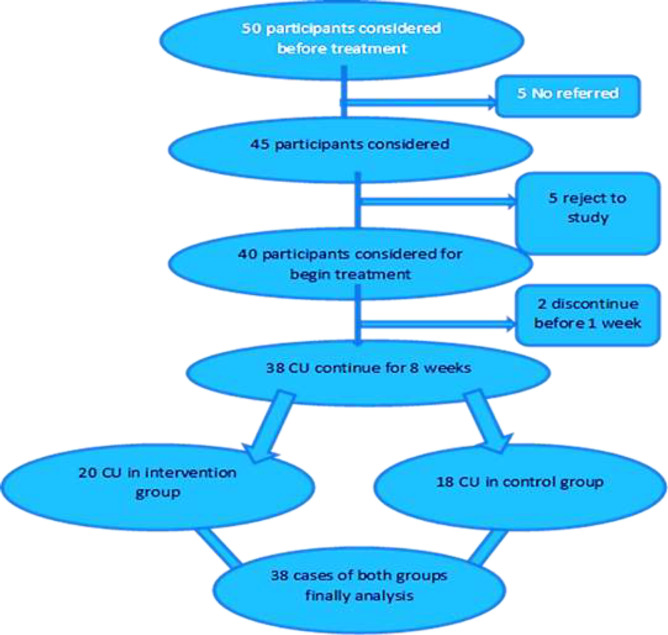
Flowchart of our study

This double-blind randomized controlled trial (RCT) was conducted between June 2019 and June 2020 (figure 1). Target population consisted of patients with CU resistant to first line treatment with cetirizine (10mg/day) who were referred to Tooba Clinic and Bouali Hospital in Sari in the North of Iran. An allergist and clinical immunologist visited the patients before the start of study, and again at 4 and 8 weeks after the start of treatment. Patients were divided into intervention and control groups using an online random number generator. Intervention group was given probiotics with cetirizine and control group was given placebo with cetirizine. The intervention group was given probiotics (femilact capsule, ZistTakhmir Pharmaceutical Company, Iran) plus cetirizine 20 mg/day in patients over 12 years of age or 0.5 mg/kg/day in patients under the age of 12. The control group was given placebo (Zisttakhmir Company, Iran) plus cetirizine 20 mg/day in patients over 12 years old or 0.5 mg/kg/day under 12 years of age. Femilact contains Lactobacilluses (Casei, Acidophilus, Bulgaricus and Rhamnosus), Bifidobacterium (Breve and Longum), Streptococcus Thermophilus plus peribiotic Fos. 

Our probiotic has 10^9^ colony forming units (CFU). Our placebo capsules are made up of 75% starch, 22% lactose, 1% magnesium stearate, 1% silicon dioxide and 1% talc with the same shape, size and color of the probiotic pill. The drug and the standard treatment were delivered to a pharmacy near the clinic for storage and distribution to patients. This is a four-way blind study because the physician, patients, data collector and data analyzer were not aware which group the patients belonged to. Probiotic and standard treatment pills were administered twice a day for 8 weeks. The Urticaria Activity Score for 7 days (UAS7) is a questionnaire that assesses CU symptoms, including: number of wheals and intensity of itch. This questionnaire was used to assess activity and severity of urticaria (1). Dermatology Life Quality Index (DLQI) questionnaire extracted from Finlay’s study was confirmed in Iran in Persian language by Tavakol et al. This questionnaire was used to assess the quality of life of patients (23, 24). We used the criteria for evaluating response to treatment from Nettis’ study (22): 

Total improvement (defined by a decrease of more than 90% in UAS7 score), excellent response (defined by a decrease of 30 to 90 % in UAS7 score), mild response (defined by a decrease of 10 to 30% in UAS7 score) and no response (defined by a decrease of less than 10% in UAS7 score). According to a study conducted by E. Nettis in which 28.9% of patients with refractory chronic urticaria responded appropriately to 8-week probiotic therapy,assuming the achievement of a moderate effect size (0.5) for probiotics in the treatment of refractory chronic urticaria, at a confidence interval of ninety-five percent and a study power of eighty percent using G-Power software, the minimum sample size of thirty-four people is determined. Assuming a 15% attrition rate in cases, up to forty people (twenty people in each group) can be determined (22). Inclusion criteria were patient’s consent, CU un-responsive to first line therapy of antihistamines (cetirizine 10 mg/day for ≥12 years or 0.5 mg/kg for <12 years), ages between 5 to 30 years. Exclusion criteria include intake of multivitamins and/or probiotics over the past 2 months, ages under 5 or over 30, physical urticaria, intake of antibiotic over the past 2 weeks, proven thyroid disease, evidence of Helicobacter pylori infection and other organ involvement. We did not have any run-in periods in this study. We did not observe physical activity nor dietary intake condition of our patients. The ethical code of our study was IR.MAZUMS.REC.1398.163. Our study was approved by the Iranian registry of clinical trial with registration reference of IRCT20110531006660N9.

**Statistical analysis: **Distribution of data was examined by drawing histograms and performing Kolmogorov-Smirnov test. After the confirmation of normal distribution of quantitative data, mean and standard deviation (SD) were used to describe the quantitative data. Also, frequency (percentage) was used to describe qualitative data. The mean differences of variables were evaluated using independent t-test. Also ${\mathrm{chi}}^{2}$ was used after classifying the “quality of life score” and categorizing the “clinical symptoms severity score”. Variables that were measured more than twice were analyzed by repeated measures analysis of variance (RMANOVA) and mixed between-within ANOVA after confirmation of the main assumption such as normal distribution of dependent variables, sphericity and no significant outliers. It is appreciated to note that all analyses in this RCT were based on intention-to-treat (ITT). ITT is preferred to per protocol analysis because it analyzes all of completed and near completed protocols. We used correlation analysis method for plot figure. Statistical description and analysis were done using IBM SPSS Version 25 and two-sided *p-value *≤ 0.05 was used for statistical judgment.

## Results

**Figure 2:**
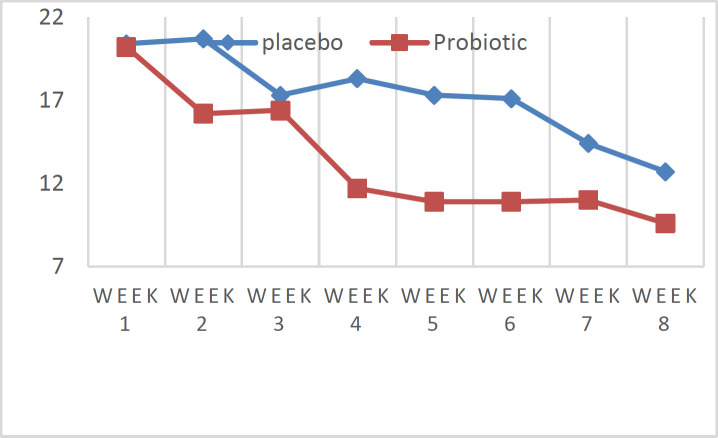
UAS7 score in groups; standard treatment and probiotic groups

Our patients aged between 7 to 30 years had a body mass index (BMI) of 15 to 38. Female and male patients made up 82% and 18%, respectively (table 1). Five patients were between the ages of 5 and 15 years. Thirty seven patients had angioedema. All patients have normal complete blood count with differential (CBC, diff), thyroid stimulating hormone (TSH), thyroxine (T4), erythrocyte sedimentation rate (ESR), C-reactive protein (CRP), aspartate-aminotransferase (AST), alanine aminotransferase (ALT), blood urea nitrogen (BUN) and serum creatinine (Cr) results. Out of all patients, two cases (one in each group) used short course oral corticosteroids (3-6 days). Three cases in each group used montelukast 10 mg/day for 1 week for control of pruritus. UAS7 scores of both groups are shown in table 2 and figure 2. The RMANOVA test results of that repeated measurements of UAS7 score in both groups during 8 weeks showed that although there is a decreasing trend, it was not statistically significant (P=0.275 in control group and 0.238 in intervention group). Also, a mixed between-within ANOVA was conducted to assess the impact of two different interventions on participant’s scores on UAS7, across 8 time periods. 

**Table 1:** Demographic data of two groups

**Variables**	**Standard treatment group**	**Intervention group**	**P-value**
Gender, N (%) Female Male	15 (83.3) 3 (16.7)	16 (80) 4 (20)	0.156*
Age, Mean (SD)	27.1±1.7	24.5±6.7	0.274^**^
BMI, Mean (SD)	27±5.7	24.1±5.7	0.284^**^

*Chi^2^test **Independent t-test

**Table 2:** UAS7 score for 8 weeks in two groups

**UAS 7 score\ group**		**Standard treatment group** **Mean ±SD**	**Intervention group** **Mean ±SD**
1st week		20.4±8.5	20.2±7.2
2^nd^ week		20.7±6.6	16.2±6.7
3rd week		17.3±6.7	16.4±11.5
4^th^ week		18.3±7.4	14.7±7.7
5^th^ week		17.3±7.7	10.9±7.6
6^th^ week		17.1±7.0	10.9±7.2
7^th^ week		14.4±7.9	11.0±6.9
8^th^ week		12.7±8.1	9.6±6.4
Repeated measures ANOVA	Wilks' lambda	0.088	0.144
	Partial Eta Squared	0.912	0.856
	P-value	0.275	0.238

The main effect comparing the two types of intervention was significant (P=0.036), suggesting the higher effectiveness of probiotic in reducing of UAS7 score during 8 weeks in comparison to standard treatment as depicted in figure 2.

**Figure 3:**
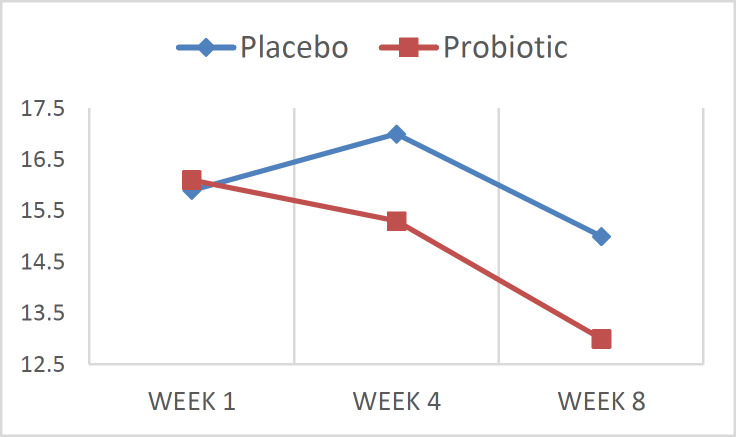
Quality of life in both groups; before, after 4- and 8-weeks treatment

Quality of life scores are shown in figures 2 and 3. Quality of life (QOL) score was 15.9±4.3 and 16.1±9.0 at baseline, 17.0±3.3 and 15.3±8.0 after 4 weeks treatment and 15.0±5.9 and 13.0±6.0 after 8 weeks treatment in control and intervention groups respectively (figure 3). 

A mixed between-within ANOVA was conducted to assess the impact of two different interventions on participant’s scores on QOL, across 3 time periods. The main effect comparing the two types of intervention was not significant (P=0.805), suggesting no effectiveness of probiotic in decreasing of QOL score during 8 weeks in comparison to standard treatment as depicted in figure 3. 

**Figure 4:**
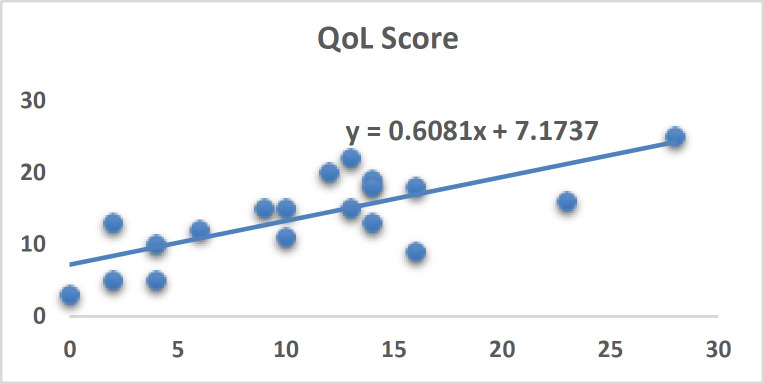
Dot plots of relation between UAS7 score and quality of life in total patients. Vertical line revealed urticaria severity and horizontal line showed quality of life in CU. There is direct relation between severities of urticaria and quality of life. There is low quality of life when severity of urticaria is higher. In this study, higher score means lower quality of life

## Discussion

Of hundreds of different microbes living in our digestive system, some of them are useful for the host’s health; these are called probiotics. Probiotics improve gastrointestinal tract microbiota, the immune system and prevent cancer (19, 25). Chronic spontaneous urticaria (CSU) is a heterogeneous skin disease that is difficult to treat. Pharmacologic therapy is often effective in the management of urticaria. The second generation of antihistamines is the first line therapy in all of urticaria including CSU (1, 2, 15). However, synthetic substances have some side effects. Other treatments of CU are H2 blockers, anti-leukotrienes, biological agents such as omalizumab and immunosuppressant such as steroids and cyclosporine (1, 2, 15-17). Therefore, we need a product for treatment of CU without side effects or with as little side effects as possible. In this regard, probiotic substances have no serious side effects (18, 19, 22). 

Demographical data (age, gender and BMI) is homogenous in both groups of our study and there were no significant differences between the two groups. More than two thirds of our patients (31 cases) were females, that is because CU is generally more common in females (1, 2, 22). We had a few children with CU, because urticaria both acute and chronic is most common between ages 20 to 50 (1, 2). In our search, we found an article studying the probiotic effects on the CU that did not include a control group (22). Our work is the only study that has a control group. In this clinical trial study, we used probiotic pearls containing 8 strains of bacteria in patients with chronic urticaria that is resistant to first line antihistamines. Our study showed that AHs are very effective in treating CU and they are mainstay and the first line pharmacological therapy in CU (1, 2). In our study UAS7 scores (CU severity) were significantly decreased after 2 months of treatment in the intervention group. Our results were the same as in Nettis’ study; they used probiotics in their patients with CU and observed resolution in 11 cases with good resolution only in two patients, although the other patients experienced mild resolution (22). 27 patients included in Nettis’ study did not show any improvements. Unfortunately, they had no control group for comparison (22). One of the reason probiotics were more effective in our study compared Nettis et al’s study is the different strains between the two studies. We used 8 strains of probiotic compared to only two strains (Lactobacillus salivarius LS01 and Bifidobacterium breve BR03) in Nettis et al’s study (22). Nettis et al. revealed more improvement in CU that is associated with allergic rhinitis (AR) than CU without AR (22). Although other factors such as hidden underlying disorders, genetic factors, allergic or non-allergic phenotype, atopy or non-atopy, duration of CU and colony count of probiotic may affect the results (26). Our study showed that the quality of life decreased in both groups but there was a no significant difference between the two groups. It is the same in Nettis’ study (22). 

However, antihistamines with or without probiotic could improve quality of life of CU patients significantly. The relationship between severity of urticaria and quality of life curve is shown in figure 4. When UAS7 score decreases, the quality of life gets better. There is a reverse relationship between severity of urticaria and quality of life. Improvement of CU by probiotics is due to modulation of the intestinal and or skin immune system (promoting TH1 cytokines such as TGF-β and IL-10and decreasing IgE) thus, probiotics relieve atopic dermatitis and dry skin (22, 27-31). Limitations of our study include small sample size, unknown patients’ dietary intake, not checking serum IgE, no skin prick test to evaluate allergens and no assessment for autoimmunity such as ASST in our patients. Our study showed adding probiotics to antihistamine can quicken the course of improvement compared to antihistamine monotherapy. We suggest to conduct more research with large sample size, using probiotics or prebiotics or symbiotics with different colony counts, different strains, relating with total serum IgE and specific IgE (skin prick test or laboratory tests) and evaluating auto immune conditions. The results of this study showed that probiotic consumption along with antihistamines may decrease the activity of urticaria but not significantly when compared to antihistamines on its own. We need more studies with larger samples to explain the effects of probiotics on CU. 
